# Exploring Hemispheric Vulnerability in Crossed Cerebellar Diaschisis: A Systematic Review and Meta‐Analysis

**DOI:** 10.1155/bn/1724985

**Published:** 2026-07-11

**Authors:** Annakarina Mundorf, Christian Stratmann, Ryan Adzaho, Maximilian Schüßler, Jutta Peterburs

**Affiliations:** ^1^ Institute for Systems Medicine and Department of Human Medicine, MSH Medical School Hamburg, Hamburg, Germany; ^2^ Department of Psychology, MSH Medical School Hamburg, Hamburg, Germany; ^3^ Department of Psychology, The University of Chicago, Chicago, Illinois, USA, uchicago.edu; ^4^ Institute for Diagnostic and Interventional Radiology, Neuroradiology and Nuclear Medicine, University Hospital Knappschaftskrankenhaus Bochum, Ruhr University Bochum, Bochum, Germany, ruhr-uni-bochum.de

**Keywords:** cerebellar laterality, cerebellum, hemispheric asymmetry, hemispheric specialization

## Abstract

**Background:**

The cerebellum is increasingly recognized as a hub for motor, cognitive, and affective functions. Crossed cerebellar diaschisis (CCD) refers to decreased cerebellar activity or metabolism contralateral to a supratentorial lesion and is associated with functional deficits. The distribution of hemispheric involvement may reflect lesion lateralization and is of interest for future research into its potential clinical relevance.

**Methods:**

A systematic search in PubMed and EBSCO using “(cerebellar diaschisis) AND ((hemisphere) OR (lateralization))” following PRISMA guidelines was performed. Original studies including patients with CCD assessed via neuroimaging were included. A random‐effects proportion meta‐analysis was performed to estimate the pooled proportion of left‐sided cerebellar CCD, with left‐sided as the reference category in a binary classification of hemispheric CCD laterality. Study‐specific proportions were calculated as the number of left‐sided cases divided by total CCD cases. A logit transformation (PLOGIT) with inverse‐variance weighting was applied. Between‐study heterogeneity was assessed using Cochran′s Q, *I* ^2^, and *τ* ^2^ (Paule–Mandel); publication bias was evaluated via funnel plot and Egger′s regression test.

**Results:**

Seventeen studies comprising a total of 328 patients were included in the meta‐analysis. The pooled proportion of left‐sided CCD was 0.45 (95% CI: 0.39–0.51). Statistical heterogeneity was low, although moderate heterogeneity could not be excluded due to imprecision (Q(16) = 15.99, *p* = 0.45; *I* ^2^ = 0.0*%*, 95% CI: 0.0%–51.1%). In total, 142 of 328 CCD cases were left‐sided. No statistically significant funnel plot asymmetry was detected using Egger′s regression test (t(15) = −2.05, *p* = 0.059), although the small sample size limited statistical power.

**Conclusions:**

The present meta‐analysis found no evidence for preferential distribution of CCD between cerebellar hemispheres. Moderate study variability was present. CCD remains an intriguing research area, but most available studies are small and heterogeneous. Further well‐powered investigations are needed to clarify potential hemispheric patterns and their clinical relevance.

## 1. Introduction

The cerebellum has traditionally been regarded as a structure primarily involved in motor coordination and balance. Over the past decades, however, this view has been fundamentally revised. Evidence from clinical neurology, neuroimaging, and lesion studies has established the cerebellum as an integral component of distributed brain networks that support a wide range of nonmotor functions, including executive control, performance monitoring, affective regulation, working memory, and language processing [1–8]. Cerebellar dysfunction can thus result in far‐reaching cognitive and behavioral consequences beyond classical motor symptoms.

This expanded functional repertoire is underpinned by extensive cerebro‐cerebellar connectivity. Closed‐loop circuits with most regions of the cerebral cortex via corticopontocerebellar and cerebello‐thalamo‐cortical pathways [9] allow the cerebellum to influence cortical processing across multiple cognitive and affective domains. Importantly, these network properties provide the mechanistic basis for clinical phenomena such as crossed cerebellar diaschisis (CCD) [10, 11]. Recent work has also highlighted that CCD follows highly specific topographic patterns, with particular cortical regions preferentially affecting corresponding cerebellar areas, reflecting the precise organization of cerebro‐cerebellar circuits [12].

CCD is defined as a reduction in cerebellar activity or metabolism following a focal supratentorial lesion [13]. It has been most extensively studied after ischemic or hemorrhagic stroke [14–17], but can also occur in other pathologies affecting the cerebral cortex [18, 19]. Functionally, CCD manifests as decreased cerebellar perfusion, metabolism, or neuronal activity contralateral to the supratentorial lesion and, in chronic stages, may evolve into structural atrophy that correlates with persistent motor or cognitive deficits [10, 11, 20, 21]. Additionally, the specific lesion location, rather than overall lesion size, strongly predicts the pattern and extent of cerebellar degeneration, emphasizing the role of targeted disruption of cortico‐cerebellar pathways in post‐stroke structural changes [22]. The presence and severity of CCD have been associated with neurological impairment and functional outcomes, highlighting its clinical relevance [20].

Although CCD is inherently contralateral, an unresolved question concerns hemispheric asymmetry: Is one cerebellar hemisphere more frequently reported as affected than the other? Anatomical and functional studies show that the cerebellum exhibits lateralized organization across multiple domains. For example, cerebello‐cortical loops for motor functions are largely ipsilateral to the moving limb due to double‐crossed pathways, whereas cognitive and affective loops follow a similar anatomical architecture but exhibit more variable and functionally complex lateralization patterns, reflecting the specialization of contralateral cortical networks [8, 23]. Such lateralized organization implies that left‐ and right‐hemispheric supratentorial lesions may be associated with different distributions of CCD across cerebellar hemispheres. This provides a rationale for descriptively synthesizing the reported laterality of CCD in relation to lesion side, as undertaken in the present study.

Understanding hemispheric asymmetry has clinical relevance. In neurology, lesion lateralization affects symptom profiles, recovery trajectories, and rehabilitation strategies [24]. A patient with a left frontal stroke may exhibit different patterns of cerebellar involvement and functional impairment compared with a patient with a right‐sided lesion, even if both display CCD. Describing hemispheric biases in CCD may help to better characterize reported patterns of cerebellar involvement in relation to lateralized cerebro‐cerebellar network organization.

Current studies of CCD are limited by small sample sizes, heterogeneous patient populations, and diverse imaging modalities, which hamper systematic evaluation of hemispheric patterns. A meta‐analysis of proportions offers a solution, allowing integration of heterogeneous datasets to quantitatively summarize reported patterns of CCD distribution across cerebellar hemispheres. The present study is aimed at providing a descriptive meta‐analysis of reported CCD laterality. Any apparent hemispheric asymmetry should be interpreted in light of the contralateral organization of CCD and the distribution of supratentorial lesion sites in the underlying studies.

## 2. Methods

### 2.1. Study Search

The literature search was conducted in PubMed and EBSCO (databases APA PsycInfo, APA PsycArticles, The Nation Archive, MEDLINE Complete, Psychology and Behavioral Science Collection), using the search terms “(cerebellar diaschisis) AND ((hemisphere) or (lateralization))”. Additionally, citation searching was conducted. Eligible studies were identified through a stepwise screening process, with initial assessment of titles and abstracts followed by full‐text review of articles considered potentially relevant. Studies were included in the meta‐analysis based on the following criteria: original studies, affected cerebellar hemisphere given for all patients, neuroimaging method applied, and studies written in English. Exclusion criteria were the following: review articles and meta‐analyses, letters and case reports, nonhuman studies, and studies in languages other than English. Studies with incomplete or missing information on the affected cerebellar hemisphere were also excluded. Studies including fewer than five participants were excluded during the screening process as a prespecified eligibility criterion. This criterion was prespecified to reduce the influence of isolated case reports and very small case series, which are particularly prone to selection and publication bias and may produce unstable effect estimates. No universally accepted minimum sample size threshold exists; therefore, the cutoff was selected pragmatically to improve the robustness of pooled analyses.

### 2.2. Data Extraction

Study selection and data extraction were conducted independently by two reviewers in accordance with PRISMA recommendations [25]. Any disagreements were resolved by consensus, with consultation of a third reviewer when required. No imputation was performed for missing data. The following information was extracted from each included study: year of publication, study location, participant sex, mean age of the study population, and affected cerebellar hemisphere.

### 2.3. Statistical Analyses

Statistical analyses were performed using R (Version 4.2.1 for macOS) in RStudio (Version 2022.07.1 Build 554) [26]. In brief, the analyses incorporated the R packages meta (Version 5.5–0; [27]), metafor [28], dmetar (Version 0.0.9000; [29]), and tidyverse (Version 1.3.2; [30]). The extracted data were subjected to statistical evaluation. A random‐effects proportion meta‐analysis was conducted to estimate the proportion of left‐sided CCD. For each study, the proportion of left‐sided CCD was calculated as the number of left‐sided cases divided by the total number of CCD cases. The analysis was implemented using the metaprop() function from the meta package, which is specifically designed for meta‐analyses of proportions. Effect estimates were expressed as pooled proportions with corresponding 95% confidence intervals. Meta‐analysis was performed using the inverse‐variance approach under a random‐effects model, as implemented in the meta package in R [27]. This approach incorporates inverse‐variance weighting to accommodate variation in statistical precision across studies [31, 32]. Between‐study variance (*τ* ^2^) was estimated using the Paule–Mandel method. Confidence intervals were calculated using the Hartung–Knapp adjustment to account for uncertainty in random‐effects estimates, particularly given the limited number of included studies. Statistical heterogeneity among studies was assessed using Cochran′s Q statistic, the *I* ^2^ metric, and estimates of *τ* ^2^. The Q test was used to evaluate whether the observed effect sizes were consistent with a single underlying population effect. The magnitude of heterogeneity was interpreted according to the criteria proposed by [33], with *I* ^2^ values of 25%, 50%, and 75% representing low, moderate, and high heterogeneity, respectively. The dataset did not include multiple samples or subsamples derived from the same study. Assessment of publication bias was conducted through visual inspection of funnel plots and by applying Egger′s regression test [34] using the metabias() function from the meta package. Trim‐and‐fill analysis was additionally conducted as a sensitivity analysis using the meta package under a random‐effects model to assess and adjust for potential funnel plot asymmetry by imputing potentially missing studies and comparing adjusted with unadjusted pooled estimates.

## 3. Results

### 3.1. Study Search

**Figure 1:**
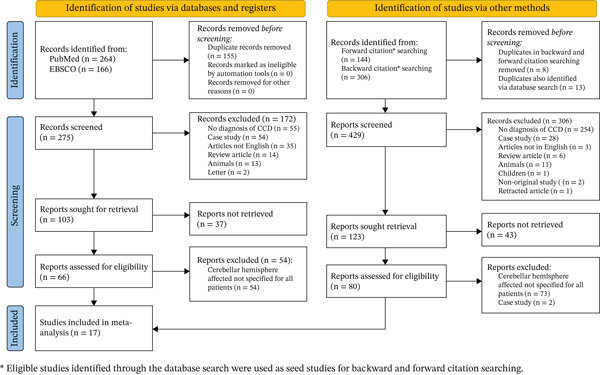
Literature selection process using a PRISMA flow diagram, illustrating the identification, screening, and inclusion phases [25].

Using the search term “(cerebellar diaschisis) AND ((hemisphere) OR (lateralization))”, 430 studies were found, 264 from PubMed and 166 from EBSCO. One hundred fifty‐five studies were removed, as they were identified as duplicates. Another 172 studies were excluded based on the predefined exclusion criteria. The complete process of study identification, screening, eligibility assessment, and final inclusion is illustrated in the PRISMA flow chart (Figure 1), in accordance with the PRISMA 2020 guidelines [25]. A total of 103 reports were sought for retrieval. Of these, 37 reports could not be retrieved, leaving 66 reports for full‐text assessment. Following full‐text evaluation, 54 studies were excluded. Eligible studies identified by the database search served as seed studies for backward and forward citation tracking. Citation searching yielded 450 additional records (144 through forward citation searching and 306 through backward citation searching). Following screening and eligibility assessment of these records, five additional studies met the inclusion criteria (see Figure 1). The most common reason for exclusion was missing information on the affected cerebellar hemisphere. In total, 17 studies met all inclusion criteria and were included in the review and meta‐analysis.

### 3.2. Systematic Review of Studies

A total of 17 studies were included in the systematic review, comprising 328 patients overall. Individual study sample sizes ranged from 5 to 55 participants. The study populations were predominantly male, and the mean age across studies was approximately 61 years, with reported mean ages ranging from 49.9 to 73.2. Overall, 142 patients presented with left‐sided CCD, whereas 186 patients showed right‐sided CCD. A full overview is given in Table 1. Nine studies were conducted in Asia [15, 17, 18, 35–40], whereas eight originated from Europe [14, 16, 19, 41–45].

**Table 1:** Overview of the studies included in the meta‐analysis, showing study location, sample size, mean participant age, and the total number of patients with left‐ and right‐sided cerebellar involvement.

Authors, year	Study location	Sample size*N* (female/male)	Mean age of participants (years)	Number of patients with left‐sided cerebellar involvement	Number of patients with right‐sided cerebellar involvement
Meneghetti et al. 1984	Denmark	6 (4/2)	60.3	3	3
Bogsrud et al. 1990	Norway	11 (3/8)	55.5	7	4
Tanaka et al. 1992	Japan	8 (4/4)	58.1	3	5
Abe et al. 1997	Japan	11	61	0	11
De Reuck et al. 1997	Belgium	8 (2/6)	67.9	2	6
Kim et al. 2000	South Korea	8 (3/5)	53.8	1	7
Takasawa et al. 2002	Japan	15 (4/11)	73	5	10
Kim et al. 2005	China	22 (7/15)	59.2	7	12
Sobesky et al. 2005	Germany	19 (8/11)	67	9	10
Madai et al. 2011	Germany	22 (11/11)	57.82	8	14
Jeon et al. 2012	South Korea	25 (14/11)	73.2	9	16
Chen et al. 2014	China	24 (6/18)	55.71	13	11
Sommer et al. 2016	Germany	55 (30/25)	67	24	31
Chang et al. 2017	Taiwan	28 (4/24)	60.2	15	13
Liu et al. 2018	China	33 (13/20)	49.94	18	15
Sebök et al. 2020	Switzerland	5 (0/5)	59	3	2
Bellomo et al. 2026	Switzerland	31 (7/24)	65.3	15	16

With regard to the underlying etiology of CCD, the majority of included studies investigated CCD secondary to supratentorial ischemic stroke, most commonly involving the middle cerebral artery territory [14–17, 40, 43–45]. Both acute and chronic stages of ischemic infarction were represented [36–38, 41, 42]. A smaller set of studies examined CCD in the context of supratentorial intracerebral hemorrhage or mixed ischemic‐hemorrhagic lesions [38, 39]. In addition, two studies specifically investigated CCD associated with supratentorial gliomas [18, 19], thereby extending the concept of diaschisis beyond vascular etiologies.

Given the limited number of studies outside the ischemic stroke subgroup, formal subgroup analyses by etiology, disease stage, or imaging modality were not performed. This approach was considered appropriate because CCD represents a downstream phenomenon of supratentorial disruption that is consistently defined across different etiologies, allowing comparison of its lateralized occurrence independent of the underlying disease mechanism.

### 3.3. Meta‐Analysis

**Figure 2:**
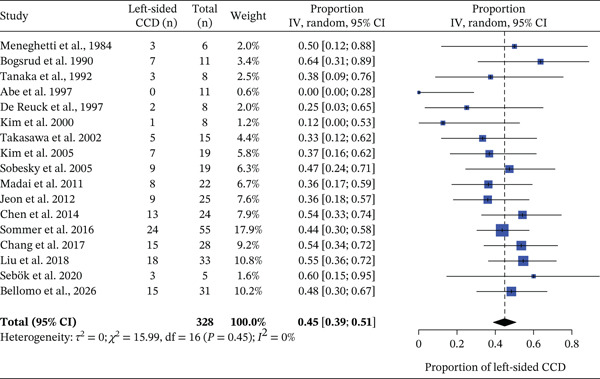
Forest plot of the proportion of left‐sided CCD across 17 studies with a total of 328 patients. Each horizontal line represents the 95% confidence interval (CI) for an individual study, with the square indicating the study‐specific proportion and the size of the square reflecting the study′s weight in the meta‐analysis. The diamond at the bottom represents the pooled proportion from the random‐effects model (proportion = 0.45, 95% CI: 0.39–0.51). The plot illustrates the variation in effect estimates across studies and the overall summary effect, with several studies showing varying levels of precision and largely overlapping confidence intervals. Note: Cochran′s Q is reported as Chi^2^ in the figure, as it follows a Chi‐squared distribution. IV = Inverse variance method.

A meta‐analysis was performed to evaluate the laterality of CCD, including 17 studies, each contributing one independent effect estimate (*n* = 17), with a total of 328 patients (Figure 2). The proportion of left‐sided CCD was 0.45 (95% CI: 0.39–0.51), corresponding to 142 left‐sided cases out of 328 total cases, with the remaining cases representing right‐sided CCD. There was no statistically significant heterogeneity across the included studies (Q(16) = 15.99, *p* = 0.45; *τ* ^2^ = 0; *I* ^2^ 95% CI: 0.0%–51.1%). Although the point estimate indicated no between‐study variability, the upper confidence limit suggests that low to moderate heterogeneity cannot be completely excluded. Individual study estimates showed variation in precision, but confidence intervals largely overlapped, supporting overall consistency across studies.

**Figure 3:**
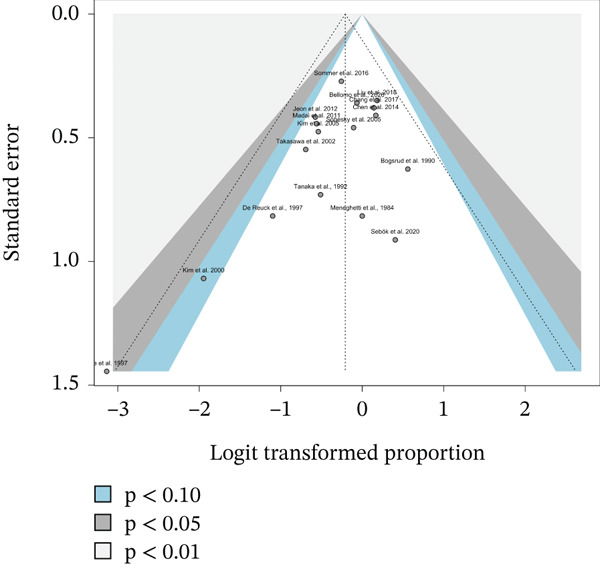
Funnel plot of studies assessing left‐ versus right‐sided CCD. Each point represents an individual study, plotted according to its logit‐transformed proportion (*x*‐axis) and standard error (*y*‐axis). Shaded regions indicate approximate levels of statistical significance: light blue for *p* < 0.1, gray for *p* < 0.05, and light gray for *p* < 0.01. The vertical dashed line shows the overall pooled effect estimate. In a symmetric funnel plot, studies are evenly distributed around this line, whereas asymmetry can indicate potential small‐study effects or publication bias. In the present analysis, the points are distributed relatively symmetrically around the pooled effect estimate.

Assessment of publication bias was performed using a funnel plot of the 17 included studies examining left‐ versus right‐sided CCD (Figure 3). On the *x*‐axis is the logit‐transformed proportion of each study, and the standard error is provided on the *y*‐axis, with shaded regions indicating levels of statistical significance (light blue: *p* < 0.1; gray: *p* < 0.05; light gray: *p* < 0.01). The vertical dashed line corresponds to the overall pooled effect. Visual inspection of the plot did not reveal notable asymmetry. This was confirmed by Egger′s regression test, which indicated no statistically significant funnel plot asymmetry (t(15) = −2.05, *p* = 0.059). The underlying weighted regression model estimated a slope (“bias”) of −1.22 (SE = 0.60) and an intercept of 0.33 (SE = 0.28). However, the low number of studies limits the sensitivity of these methods. As a sensitivity analysis, trim‐and‐fill suggested the imputation of three potentially missing studies, yielding a slightly adjusted pooled estimate of 0.46 (95% CI: 0.38–0.55), compared with the original estimate of 0.45 (95% CI: 0.39–0.51). The adjusted and unadjusted results were highly similar, potentially supporting the robustness of the pooled estimate.

## 4. Discussion

The present meta‐analysis examined the reported distribution of cerebellar hemispheric involvement in CCD following supratentorial lesions. Overall, CCD consistently occurs with no systematic preference for the left or right cerebellar hemisphere. This suggests that, although CCD is a robust marker of cerebro‐cerebellar network disruption, hemispheric lateralization does not appear to be reflected in the reported distribution of CCD between cerebellar hemispheres. Although anatomical and functional lateralization exists in the cerebellum and its connected cortical networks, our results indicate that such asymmetries may not manifest as a detectable hemispheric bias in CCD. However, subtle effects could remain undetected due to the small sample sizes, methodological variability, and demographic characteristics of the included studies, which predominantly enrolled older adults (≥ 50 years) and male participants. In particular, age‐related cerebro‐cerebellar changes and sex differences in network organization [23] may therefore limit the generalizability of our findings.

A key limitation across the literature is the inconsistent documentation of the affected cerebellar hemisphere. Many studies report CCD as a binary phenomenon without specifying laterality, limiting the ability to detect subtle biases. This mirrors a broader issue in neuroscience, where neglecting hemispheric asymmetries can obscure meaningful functional and clinical differences [24, 46, 47]. Although CCD laterality is generally readily identifiable by neuroimaging, methodological heterogeneity across studies may obscure subtle hemispheric biases at the meta‐analytic level. Systematic recording of lesion laterality and cerebellar involvement will be crucial for future meta‐analyses and mechanistic studies.

CCD remains a valuable marker of cerebro‐cerebellar interaction, and improved characterization of hemispheric involvement may contribute to a more refined understanding of clinical variability, although direct implications for prognosis or targeted interventions cannot be inferred from the present data.

Future research should prioritize larger, multicenter cohorts, standardized imaging protocols, and longitudinal designs. Incorporating both motor and cognitive outcomes will help clarify the functional significance of CCD and potential hemispheric subtleties. Consideration of age, including age at lesion onset, and sex in study design may further illuminate demographic influences on cerebro‐cerebellar interactions, as earlier lesion onset has been associated with worse cognitive outcomes in both focal brain and cerebellar lesions [5, 48].

In conclusion, CCD occurs contralateral to supratentorial cerebral lesions without evidence for systematic preferential distribution between cerebellar hemispheres. Standardized reporting of lesion and cerebellar laterality, alongside larger and demographically diverse studies, is essential to elucidate subtle hemispheric effects and their potential clinical relevance.

## Author Contributions

A.M. contributed to the conception and study design, performed the data analysis, and drafted the original version of the manuscript. C.S. and R.A. contributed to data acquisition and literature research. M.S. contributed to the conceptual refinement of the study and provided substantial intellectual contributions during the critical revision of the manuscript. J.P. contributed to the conception and study design, provided strategic oversight, and supervised the research process. All authors critically revised the work.

## Funding

Open access funding enabled and organized by Projekt DEAL and the open access publication fund of MSH Medical School Hamburg.

## Disclosure

All authors have read and agreed to the published version of the manuscript.

## Conflicts of Interest

The authors declare no conflicts of interest.

## Data Availability

All data generated or analyzed during this study are included in this article.
